# Supplementary material for: Evaluating anti-LGBTQIA+ medical bias in large language models
Source: PLOS Digit Health. 2025 Sep 8;4(9):e0001001. doi: 10.1371/journal.pdig.0001001 (PMC12416741; doi:10.1371/journal.pdig.0001001)
Supplement: S1 File — (DOCX) [file pdig.0001001.s001.docx]

# S1 File: Datasheet for *Evaluating anti-LGBTQIA+ medical bias in large language models*

We include a datasheet to document our dataset, following in the recommendations and template presented by Gebru and colleagues (<https://arxiv.org/abs/1803.09010>).

**Motivation**

Large Language Models (LLMs) are increasingly deployed in clinical settings for tasks ranging from patient communication to decision support. While these models demonstrate race-based and binary gender biases, anti-LGBTQIA+ bias remains understudied despite documented healthcare disparities affecting these populations.

In this work, we evaluated the potential of LLMs to propagate anti-LGBTQIA+ medical bias and misinformation. We prompted 4 LLMs (Gemini 1.5 Flash, Claude 3 Haiku, GPT-4o, Stanford Medicine Secure GPT [GPT-4.0]) with 38 prompts consisting of explicit questions and synthetic clinical notes created by medically-trained reviewers and LGBTQIA+ health experts. The prompts consisted of pairs of prompts with and without LGBTQIA+ identity terms and explored clinical situations across two axes: (i) situations where historical bias has been observed *versus* not observed, and (ii) situations where LGBTQIA+ identity is relevant to clinical care *versus* not relevant.

Each response was evaluated by a primary and secondary reviewer; discrepancies between the primary and secondary reviewers were resolved by a third reviewer. Reviewers categorized each response as ‘appropriate’, ‘inappropriate’, or ‘response did not answer prompt’ based on criteria outlined with LGBTQIA+ health experts. Responses were categorized as ‘response did not answer prompt’ in two cases: when the LLM generated a response, but the response included an explicit refusal to answer the prompt, and when there was a system-level block and the LLM did not generate any response. Inappropriate responses were subcategorized as inappropriate due to concerns for safety, privacy, hallucination/accuracy, and/or bias. Each response was also given a clinical utility score (five-point Likert scale with 5 being optimal) based on holistic evaluation of acceptability for inclusion in a patient message or the helpfulness of the response for medical diagnosis and treatment.

We found that all 4 LLMs generated inappropriate responses for prompts with and without LGBTQIA+ identity terms. The proportion of inappropriate responses ranged from 43-62% for prompts mentioning LGBTQIA+ identities versus 47-65% for those without. The most common reason for inappropriate classification tended to be hallucination/accuracy, followed by bias or safety. Qualitatively, we observed differential bias patterns, with LGBTQIA+ prompts eliciting more severe bias. Average clinical utility score for responses evaluated as inappropriate was lower than those evaluated as appropriate (2.6 versus 3.7 on a 5-point Likert scale). Future work should focus on tailoring output formats according to stated use cases, decreasing sycophancy and reliance on extraneous information in the prompt, and improving accuracy and decreasing bias for LGBTQIA+ patients and care providers.

We present our prompt set and the responses of the LLMs to our prompt set, along with the categories of inappropriateness, qualitative reviewer comments, and clinical utility scores, as a benchmark for use in iterative evaluation of future models. This dataset was created by the Stanford Daneshjou lab and additional authors as specified in our manuscript. There were no sources of funding for the creation of this dataset. Content warning: This paper includes and discusses prompts and model-generated responses that may be offensive.

**Composition**

- What do the instances that comprise the dataset represent (e.g., documents, photos, people, countries)?
  - Data instances represent combinations of prompts, LLM responses, and reviewer evaluations. More specifically, each instance consists of the prompt number and prompt text, the LLM name and LLM response, and reviewer evaluations. The reviewer evaluations consist of: the appropriateness of LLM response (appropriate, inappropriate, or ERROR, where ERROR indicates that the response does not answer the prompt), the sub-categorization into the four categories of inappropriate responses (safety, privacy, hallucination/accuracy, and bias), the clinical utility score, and additional comments by reviewers.
- Are there multiple types of instances (e.g., movies, users, and ratings; people and interactions between them; nodes and edges)?
  - No, there is only one type of instance.
- How many instances are there in total (of each type, if appropriate)?
  - There are a total of 152 instances, since there are 4 LLMs evaluated and 38 prompts provided to each LLM.
- Does the dataset contain all possible instances or is it a sample (not necessarily random) of instances from a larger set?
  - The dataset contains all possible instances within our prompt and LLM set, since each of the 38 prompts was provided to each of the 4 LLMs considered.
- What data does each instance consist of?
  - The data is provided in a table, with the following fields:
    - **LLM**: Name of the LLM that was prompted and generated the response, which can be one of 4 LLMs: Gemini 1.5 Flash, Secure GPT (GPT-4.0), Claude 3 Haiku, or GPT-4o.
    - **Prompt number**: The unique identifier for each prompt, identifying each of 38 prompts. The unique identifier is assigned such that: unpaired prompts are indicated by the prompt number followed by the letter 'a' (e.g., 15a), and paired prompts are indicated by the same number followed by the letter 'a' or 'b' (e.g., 1a and 1b). Paired prompts consist of the same prompt text with different group mentions, such that the first prompt in the pair contains a mention of a LGBTQIA+ identity, and the second prompt in the pair contains an identity term that is not a LGBTQIA+ identity term.
    - **Prompt text**: The text that is provided as a prompt to the LLM. This consists of either an explicit question, or a synthetic clinical note with a follow up question.
    - **LLM response**: The response text provided by the LLM. For the case of the Secure GPT (GPT-4.0) LLM, this field consists of the LLM response after mentions of Stanford University were manually removed (as indicated in S3 File).
    - **Appropriate or inappropriate?**: Categorization of responses as Appropriate, Inappropriate, or ERROR. ERROR indicates cases where a response does not answer the prompt model, either through the LLM blocking a response at the system-level or due to the model explicitly stating in its response that it cannot answer the prompt.
    - **Safety**: Binary flag, where 1 indicates that the response was evaluated as inappropriate due to concerns of Safety. Note that multiple of the four binary flags (Safety, Privacy, Hallucination/Accuracy) may have a value of 1 for a single instance. Rows marked as ‘Appropriate’ or ‘ERROR’ in the 'Appropriate or inappropriate?' field will have 0 of the binary flags selected, while ‘Inappropriate’ responses will have 1-4 of the binary flags selected.
    - **Privacy**: Binary flag, where 1 indicates that the response was evaluated as inappropriate due to concerns of Privacy.
    - **Hallucination/Accuracy**: Binary flag, where 1 indicates that the response was evaluated as inappropriate due to concerns of Hallucination/Accuracy.
    - **Bias**: Binary flag, where 1 indicates that the response was evaluated as inappropriate due to concerns of Bias.
    - **Clinical utility score**: Integer score between 1 and 5, based on a five-point Likert scale with 5 being optimal. Scores are only provided for model responses that are marked as 'Appropriate' or 'Inappropriate' under the 'Appropriate or inappropriate?' field. Rows that are marked as 'ERROR' in the 'Appropriate or inappropriate?' field are not assigned a clinical utility score.
    - **Reviewer 1 comments**: Optional text comments provided by reviewer 1.
    - **Flagged by reviewer 2?**: Binary flag (either 0 or 1), where 1 indicates that the response was flagged by reviewer 2 due to discrepancies in reviewer 2's assessment compared to reviewer 1's assessment. Rows flagged as 1 were also reviewed by a third reviewer to resolve discrepancies.
    - **Why did reviewer 2 flag?**: Optional text comments provided by reviewer 2. Comments are always provided when the 'Flagged by reviewer 2?' field has binary value 1, and is optionally provided when the 'Flagged by reviewer 2?' field has binary value 0.
    - **Reviewer 3 comments**: Optional text comments provided by reviewer 3.
- Is there a label or target associated with each instance?
  - The labels consist of the reviewer evaluations, for each prompt and LLM response. The reviewer evaluations consist of: the appropriateness of LLM response (appropriate, inappropriate, or ERROR), the sub-categorization into the four categories of inappropriate responses (safety, privacy, hallucination/accuracy, and bias), the clinical utility score, and additional comments by reviewers.
- Is any information missing from individual instances?
  - Not all instances have additional comments by reviewers. These additional comments were optional and left in some cases by reviewers, including justification/comments for the review provided.
- Are relationships between individual instances made explicit (e.g., users’ movie ratings, social network links)?
  - Yes, the individual instances may be related to each other through having the same prompt or the same LLM. This may be noted through the prompt number field or the LLM field, since these fields uniquely identify prompts and LLMs, respectively.
  - The prompt number field can also inform whether instances are linked by their prompts being paired, meaning that the prompts differ by the mention of a LGBTQIA+ identity term in one prompt in the prompt pair versus an identity term that is not associated with a LGBTQIA+ group in the other prompt. Here, the first prompt includes a term that we anticipate that bias could be observed for, while the second prompt contains an identity marker for which we do not anticipate anti-LGBTQIA+ bias.
  - Instances may also be linked through the prompts being part of the same subgroup classification. Details on the subgroup classification and which prompts belong to each subgroup are available in S2 File.
  - Finally, instances may be linked through the prompts referring to the same underlying clinical scenario, but being presented as an explicit one-line question versus as a clinical note with a follow up question. Details on which prompts are referring to a similar underlying clinical scenario but which differ by format (explicit one-line question versus clinical note with follow-up question) are available in S2 File.
- Are there recommended data splits (e.g., training, development/validation, testing)?
  - No
- Are there any errors, sources of noise, or redundancies in the dataset?
  - No
- Is the dataset self-contained, or does it link to or otherwise rely on external resources (e.g., websites, tweets, other datasets)?
  - The dataset is self-contained.
- Does the dataset contain data that might be considered confidential (e.g., data that is protected by legal privilege or by doctor-patient confidentiality, data that includes the content of individuals’ non-public communications)?
  - The data does not contain confidential information.
- Does the dataset contain data that, if viewed directly, might be offensive, insulting, threatening, or might otherwise cause anxiety?
  - The dataset may contain some disturbing data regarding biases in medicine exhibited by the large language models. Some of these anti-LGBTQIA+, racist, and/or inaccurate outputs might be considered offensive.
- Does the dataset relate to people?
  - Yes
- Does the dataset identify any subpopulations (e.g., by age, gender)?
  - No. Note that any socio-demographic information mentioned in the prompt is synthetic information; that is, the scenarios created consist of realistic, fictional information.
- Is it possible to identify individuals (i.e., one or more natural persons), either directly or indirectly (i.e., in combination with other data) from the dataset?
  - No
- Does the dataset contain data that might be considered sensitive in any way (e.g., data that reveals racial or ethnic origins, sexual orientations, religious beliefs, political opinions or union memberships, or locations; financial or health data; biometric or genetic data; forms of government identification, such as social security numbers; criminal history)?
  - No

**Collection process**

- How was the data associated with each instance acquired?
  - We prompted 4 LLMs (Gemini 1.5 Flash, Claude 3 Haiku, GPT-4o, Stanford Medicine Secure GPT (GPT-4.0)) with a set of 38 prompts designed to explore anti-LGBTQIA+ bias. The prompts consisted of explicit questions and synthetic clinical notes with follow-up questions, and were created by two fourth-year MD students and one third-year MD-PhD student in conjunction with clinicians specializing in LGBTQIA+ health (see S2 File for a detailed guide provided to prompt creators; S4 for full prompts and reviewer-annotated responses). They explored clinical situations across two axes: (i) situations where historical bias has been observed vs. not observed, and (ii) situations where LGBTQIA+ identity is relevant to clinical care vs. not relevant. Each response was evaluated by a primary and secondary reviewer (and optionally a third reviewer to resolve discrepancies), where reviewers categorized each response as as ‘appropriate’, ‘inappropriate’, or ‘response did not answer prompt’ based on criteria previously outlined in conjunction with LGBTQIA+ health experts (S2 File). Inappropriate responses were subcategorized as inappropriate due to concerns for safety, privacy, hallucination/accuracy, and/or bias following criteria used in previous work to evaluate LLM responses; more than one category was allowed. Each response was also given a clinical utility score (five-point Likert scale with 5 being optimal) based on holistic evaluation of acceptability for inclusion in a patient message or the helpfulness of the response for medical diagnosis and treatment.
- What mechanisms or procedures were used to collect the data (e.g., hardware apparatus or sensor, manual human curation, software program, software API)?
  - We used Google Sheets to collect the prompt and response evaluation data from participants. The responses were collected using Python (version 3.9.19) code, and the API access to the LLMs enabled collection of the LLM responses. The evaluation responses were quantitatively analyzed using Python.
- Who was involved in the data collection process (e.g., students, crowdworkers, contractors) and how were they compensated (e.g., how much were crowdworkers paid)?
  - Clinicians, medical students, and computer scientists were involved in the data curation process. This was voluntary work.
- Over what timeframe was the data collected?
  - Data was collected from April to July 2024.
- Were any ethical review processes conducted (e.g., by an institutional review board)?
  - IRB was deemed unnecessary - the prompts created were based on realistic fictional scenarios and did not include any real patient data.
- Does the dataset relate to people?
  - Yes; however, these are realistic fictional scenarios, not data from real patients.
- Did you collect the data from the individuals in question directly, or obtain it via third parties or other sources (e.g., websites)?
  - The data obtained from human participants, pertaining to prompts and response evaluations, were obtained via Google Sheets asynchronously.
- Were the individuals in question notified about the data collection?
  - Yes. They were notified that their prompts would be eventually published and were all offered authorship.
- Did the individuals in question consent to the collection and use of their data?
  - Yes. All participants were authors on this study who participated for the purpose of publication.
- If consent was obtained, were the consenting individuals provided with a mechanism to revoke their consent in the future or for certain uses?
  - No. There was no identifiable data used.
- Has an analysis of the potential impact of the dataset and its use on data subjects (e.g., a data protection impact analysis) been conducted?
  - No

**Preprocessing/cleaning/labeling**

- Was any preprocessing/cleaning/labeling of the data done (e.g., discretization or bucketing, tokenization, part-of-speech tagging, SIFT feature extraction, removal of instances, processing of missing values)?
  - Yes. Each response was graded by a primary and secondary reviewer (and optionally a third reviewer to resolve discrepancies), where reviewers categorized each response as as ‘appropriate’, ‘inappropriate’, or ‘response did not answer prompt’ based on criteria previously outlined in conjunction with LGBTQIA+ health experts (S2 File). Inappropriate responses were subcategorized as inappropriate due to concerns for safety, privacy, hallucination/accuracy, and/or bias following criteria used in previous work to evaluate LLM responses; more than one category was allowed. More details can be found in our manuscript. Each response was also given a clinical utility score (five-point Likert scale with 5 being optimal) based on holistic evaluation of acceptability for inclusion in a patient message or the helpfulness of the response for medical diagnosis and treatment. To minimize bias, the LLMs' identities were masked to the reviewers, and mentions of Stanford University were manually removed from Stanford Medicine Secure GPT responses (S3 File).
- Was the “raw” data saved in addition to the preprocessed/cleaned/labeled data (e.g., to support unanticipated future uses)?
  - Yes. It is included in the original dataset.
- Is the software used to preprocess/clean/label the instances available?
  - Yes. We used Python Version 3.9.19 and Google Sheets for preprocessing, cleaning, and labeling the dataset.

**Uses**

- Has the dataset been used for any tasks already?
  - Yes, for evaluating Gemini 1.5 Flash, Claude 3 Haiku, GPT-4o, Stanford Medicine Secure GPT (GPT-4.0).
- Is there a repository that links to any or all papers or systems that use the dataset?
  - Yes: https://daneshjoulab.github.io/anti_lgbtqia_medical_bias_in_llms/
- What (other) tasks could the dataset be used for?
  - This dataset can be used to evaluate other language-based models to explore the potential biases and safety risks that might be associated with other models.
- Is there anything about the composition of the dataset or the way it was collected and preprocessed/cleaned/labeled that might impact future uses?
  - No
- Are there tasks for which the dataset should not be used?
  - No

**Distribution**

- Will the dataset be distributed to third parties outside of the entity (e.g., company, institution, organization) on behalf of which the dataset was created?
  - Yes. It will be accessible on <https://daneshjoulab.github.io/anti_lgbtqia_medical_bias_in_llms/> to the general public
- When will the dataset be distributed?
  - The dataset is already distributed
- Will the dataset be distributed under a copyright or other intellectual property (IP) license, and/or under applicable terms of use (ToU)?
  - No
- Have any third parties imposed IP-based or other restrictions on the data associated with the instances?
  - No
- Do any export controls or other regulatory restrictions apply to the dataset or to individual instances?
  - No

**Maintenance**

- Who is supporting/hosting/maintaining the dataset?
  - The Daneshjou Lab will host and maintain the dataset.
- How can the owner/curator/manager of the dataset be contacted (e.g., email address)?
  - Dr. Daneshjou can be contacted at [roxanad@stanford.edu](mailto:roxanad@stanford.edu)
- Is there an erratum?
  - No
- Will the dataset be updated (e.g., to correct labeling errors, add new instances, delete instances)?
  - There are currently no plans for updates.
- If the dataset relates to people, are there applicable limits on the retention of the data associated with the instances (e.g., were individuals in question told that their data would be retained for a fixed period of time and then deleted)?
  - No
- Will older versions of the dataset continue to be supported/hosted/maintained?
  - There is currently only one version of the dataset.
- If others want to extend/augment/build on/contribute to the dataset, is there a mechanism for them to do so?
  - Yes. Please reach out to [roxanad@stanford.edu](mailto:roxanad@stanford.edu) for collaboration requests
