# Supplementary material for: Evaluating anti-LGBTQIA+ medical bias in large language models
Source: PLOS Digit Health. 2025 Sep 8;4(9):e0001001. doi: 10.1371/journal.pdig.0001001 (PMC12416741; doi:10.1371/journal.pdig.0001001)
Supplement: S3 File — (DOCX) [file pdig.0001001.s003.docx]

# S3 File: Methods for removing mentions of Stanford University/Stanford Medicine from Secure GPT responses

In our reviewing strategy, we sought to minimize bias that could be caused due to preconceived notions regarding which LLMs may be more biased/inaccurate. Toward this, we masked the identities of the LLMs to the reviewers during the response reviewing process. For the responses obtained from Stanford Medicine’s Secure GPT, there were responses (5 responses out of 38 total responses) that had mentions of Stanford, Stanford Health Care, and/or Stanford School of Medicine. For these responses, we removed these mentions prior to assessment by the reviewers, in order to maintain the masking of LLM identities to the reviewers. For the five prompts that were edited to remove mentions, the edit involved a straightforward removal of the phrase including the mention (for responses to prompts 3a, 7b, and 21a), replacing the mention with a placeholder for a name (for response to prompt 20b), or editing the phrase from active voice to passive voice (for response to prompt 21b) (Table A). In using this methodology, we assume that the sections of replaced text (including mentions of Stanford, Stanford Health Care, and/or Stanford School of Medicine) are unrelated to the reviewer task and thus have no impact on reviewer assessment.

**Table A. Instances of replacing mentions of Stanford University/Stanford Medicine from Secure GPT’s responses.**

| **Prompt Number** | **Excerpt from model response (with mention of Stanford University/Stanford Medicine)** | **Excerpt from edited model response (to remove mention of Stanford University/Stanford Medicine)** |
| --- | --- | --- |
| 3a | “As an AI developed by Stanford” | “As an AI” |
| 7b | “I'm an AI developed by researchers at Stanford” | “I'm an AI” |
| 20b | “Sincerely,  Stanford Health Care and Stanford School of Medicine.” | “Sincerely,  [Your Name]” |
| 21a | “Sincerely,  [Your Name]  [Your Title]  Stanford Health Care and Stanford School of Medicine” | “Sincerely,  [Your Name]  [Your Title]” |
| 21b | “Stanford Health Care offers various smoking cessation resources and supports” | “Various smoking cessation resources and supports are offered” |
