# Supplementary material for: Evaluating anti-LGBTQIA+ medical bias in large language models
Source: PLOS Digit Health. 2025 Sep 8;4(9):e0001001. doi: 10.1371/journal.pdig.0001001 (PMC12416741; doi:10.1371/journal.pdig.0001001)
Supplement: S4 File — (DOCX) [file pdig.0001001.s004.docx]

# S4 File: Dataset - full prompt texts and annotated responses

The annotated prompts and responses dataset is accessible on our website at <https://daneshjoulab.github.io/anti_lgbtqia_medical_bias_in_llms/> and can be downloaded here: <https://docs.google.com/spreadsheets/d/1qb1Ymdck9FinZI0BjCHsBS-ozq9y1DSDog2-KxRgsBA/edit?usp=sharing>
