# Supplementary material for: Evaluating anti-LGBTQIA+ medical bias in large language models
Source: PLOS Digit Health. 2025 Sep 8;4(9):e0001001. doi: 10.1371/journal.pdig.0001001 (PMC12416741; doi:10.1371/journal.pdig.0001001)
Supplement: S5 File — (DOCX) [file pdig.0001001.s005.docx]

# S5 File: Quantitative result tables

**Table A. Counts and percentages of LLM responses that were categorized as appropriate, inappropriate or the response did not answer the prompt, for each LLM and identity mention type. Note: The total number of prompts with LGBTQIA+ identity mentioned is greater than the number of prompts without LGBTQIA+ identity mentioned (21 *versus* 17 prompts).**

| **LLM** | **Identity Mention Type** | **Appropriate or inappropriate?** | | |
| --- | --- | --- | --- | --- |
|  |  | **Appropriate** | **Inappropriate** | **Response did not answer prompt** |
| ***Gemini 1.5 Flash*** | **LGBTQIA+ identity mentioned** | 4 (19.0%) | 10 (47.6%) | 7 (33.3%) |
|  | **Not mentioned** | 4 (23.5%) | 8 (47.1%) | 5 (29.4%) |
| ***Claude 3 Haiku*** | **LGBTQIA+ identity mentioned** | 5 (23.8%) | 13 (61.9%) | 3 (14.3%) |
|  | **Not mentioned** | 6 (35.3%) | 11 (64.7%) | 0 (0.0%) |
| ***GPT-4o*** | **LGBTQIA+ identity mentioned** | 9 (42.9%) | 12 (57.1%) | 0 (0.0%) |
|  | **Not mentioned** | 9 (52.9%) | 8 (47.1%) | 0 (0.0%) |
| ***Stanford Medicine Secure GPT (GPT-4.0)*** | **LGBTQIA+ identity mentioned** | 12 (57.1%) | 9 (42.9%) | 0 (0.0%) |
|  | **Not mentioned** | 7 (41.2%) | 10 (58.8%) | 0 (0.0%) |

**Table B. Counts and percentages of each inappropriate subcategory, for prompts with LGBTQIA+ identity mentioned versus not mentioned. The percentages indicate the percentage of inappropriate responses for each LLM and identity mention type that were categorized into each inappropriate response subcategory. Note: the total number of prompts with LGBTQIA+ identity mentioned is greater than the number of prompts without LGBTQIA+ identity mentioned (21 *versus* 17 prompts). Also, the percentages in each row need not add up to 100%, since each inappropriate response could be categorized into more than one subcategory.**

| **LLM** | **Identity Mention Type** | **Category of Inappropriate Response** | | | |
| --- | --- | --- | --- | --- | --- |
|  |  | **Safety** | **Privacy** | **Hallucination/Accuracy** | **Bias** |
| ***Gemini 1.5 Flash*** | **LGBTQIA+ identity mentioned** | 5 (50.0%) | 0 (0.0%) | 5 (50.0%) | 4 (40.0%) |
|  | **Not mentioned** | 2 (25.0%) | 0 (0.0%) | 6 (75.0%) | 2 (25.0%) |
| ***Claude 3 Haiku*** | **LGBTQIA+ identity mentioned** | 2 (15.4%) | 0 (0.0%) | 12 (92.3%) | 5 (38.5%) |
|  | **Not mentioned** | 2 (18.2%) | 0 (0.0%) | 10 (90.9%) | 2 (18.2%) |
| ***GPT-4o*** | **LGBTQIA+ identity mentioned** | 2 (16.7%) | 0 (0.0%) | 11 (91.7%) | 5 (41.7%) |
|  | **Not mentioned** | 0 (0.0%) | 0 (0.0%) | 7 (87.5%) | 3 (37.5%) |
| ***Stanford Medicine Secure GPT (GPT-4.0)*** | **LGBTQIA+ identity mentioned** | 1 (11.1%) | 0 (0.0%) | 7 (77.8%) | 6 (66.7%) |
|  | **Not mentioned** | 1 (10.0%) | 0 (0.0%) | 7 (70.0%) | 5 (50.0%) |

**Table C. Average and standard deviation of clinical utility score for each LLM, for responses categorized as appropriate versus responses categorized as inappropriate.**

| **LLM** | **Average Clinical Utility Score** | | **Standard Deviation of Clinical Utility Score** | |
| --- | --- | --- | --- | --- |
|  | **Appropriate responses** | **Inappropriate responses** | **Appropriate responses** | **Inappropriate responses** |
| ***Gemini 1.5 Flash*** | 3.00 | 2.67 | 1.07 | 0.69 |
| ***Claude 3 Haiku*** | 3.09 | 2.38 | 0.83 | 0.88 |
| ***GPT-4o*** | 4.39 | 2.85 | 0.70 | 0.81 |
| ***Stanford Medicine Secure GPT (GPT-4.0)*** | 3.79 | 2.68 | 0.71 | 0.58 |
| ***All Models*** | 3.73 | 2.63 | 0.94 | 0.77 |
